# Risk factors and morbidity characteristics of surgical site infection following ankle fracture ORIF: A comprehensive analysis

**DOI:** 10.1097/MD.0000000000051389

**Published:** 2026-11-06

**Authors:** Chenyang Zhao, Xiaobo Fan, Chaojian Pang

**Affiliations:** a Department of Trauma Orthopedics, Handan First Hospital, China.

**Keywords:** ankle fracture, morbidity characteristics, risk factors, surgical site infection

## Abstract

This study aimed to describe the incidence and microbiological characteristics of surgical site infection (SSI) after open reduction and internal fixation (ORIF) of ankle fractures and identify clinical factors associated with SSI. This single-center retrospective cohort study included 248 patients who underwent ankle fracture ORIF between January and December 2023. SSI occurring within 30 postoperative days was identified from available inpatient and postoperative medical records. Demographic, injury-related, laboratory, and perioperative variables were compared between patients with and without SSI. Multivariable logistic regression was used to estimate adjusted associations with SSI. SSI occurred in 32 of 248 patients (12.9%), including 25 superficial and 7 deep infections. Cultured organisms included Escherichia coli (n = 10), Staphylococcus aureus (n = 7), Klebsiella pneumoniae (n = 6), Enterobacter cloacae (n = 5), and Staphylococcus epidermidis (n = 4). In multivariable analysis, open fracture, wound class II–III, and neutrophil percentage > 75% were associated with SSI. Age and body mass index (BMI) also showed positive associations, although the precise unit-specific effect sizes could not be reliably reconstructed from the archived statistical analysis; these comparatively large per-unit estimates should be interpreted cautiously because of the limited number of SSI events and potential residual confounding. SSI occurred in 12.9% of this single-center ankle fracture cohort. Older age and higher BMI showed associations with SSI, together with open fracture, less-clean operative wounds, and neutrophilia. The magnitude of the age- and BMI-related effects should be interpreted cautiously because their original regression scaling could not be independently reconstructed.

## 1. Introduction

Ankle fractures are common orthopedic injuries and account for a substantial proportion of lower-extremity fractures. Unstable and displaced injuries are frequently treated with open reduction and internal fixation to restore ankle congruity and mechanical stability. Despite improvements in fixation techniques and perioperative care, surgical site infection remains an important complication because it can delay wound and fracture healing, prolong hospitalization, increase antimicrobial exposure, and lead to repeated surgery.^[1,2]^

Reported infection rates after ankle fracture surgery vary widely because previous studies have differed in study population, inclusion of open fractures, definitions of superficial and deep infection, and surveillance duration. Large cohorts and systematic reviews have identified open fracture, smoking, diabetes, advanced age, obesity, higher American Society of Anesthesiologists classification, and wound contamination as relevant risk domains. Local microbiological information may additionally support empirical antimicrobial decisions and targeted prevention.^[3,4]^

The objectives of this study were to characterize SSI occurring within 30 days after ankle fracture ORIF, describe the cultured pathogens, and evaluate clinical and perioperative factors associated with infection.

## 2. Information and methodology

### 2.1. General information

This retrospective cohort study was conducted at Handan First Hospital and included patients treated from January through December 2023. The study was approved by the institutional medical ethics committee and was conducted in accordance with the ethical principles applicable to retrospective clinical research. A total of 248 patients with ankle fractures who underwent open reduction and internal fixation were included, comprising 142 men (57.3%) and 106 women (42.7%). The patients ranged in age from 42 to 71 years, with a mean age of 48.26 ± 3.89 years. body mass index (BMI) ranged from 22 to 31 kg/m2, with a mean of 24.39 ± 1.30 kg/m2. The time from fracture to surgery ranged from 1 to 8 days, with a mean of 4.27 ± 1.04 days. Operative duration ranged from 72 to 188 minutes, with a mean of 140.54 ± 10.05 minutes. Intraoperative blood loss ranged from 105 to 220 mL, with a mean of 158.73 ± 9.62 mL. The length of hospital stay ranged from 9 to 28 days, with a mean of 17.35 ± 2.70 days.

### 2.2. Inclusion and exclusion criteria

Inclusion criteria were: ankle fracture confirmed by imaging; an indication for ORIF; and documented informed consent. Exclusion criteria were: old or pathological fracture; a documented blood-circulation disorder or major organ dysfunction; and preoperative acute or chronic infection or neuromuscular dysfunction.

### 2.3. Methods

All patients were operated with open reduction and internal fixation. All patients received standard perioperative antibiotic prophylaxis as per hospital regulations. They were administered cefazolin. If the patients were allergic to cefazolin, clindamycin was used instead. Imaging examination was performed to assess the fracture and determine the surgical plan. The physician instructed the patient to maintain a lying position, performed epidural anesthesia combined with subarachnoid anesthesia, placed a pneumatic tourniquet in the appropriate position of the lower limb, and cleaned and disinfected the fracture site. If the patient had an external ankle fracture, the posterior tibial margin was chosen to set up the surgical incision; if the patient had an internal ankle fracture, the anterior side of the internal ankle was chosen to set up the surgical incision. The fracture ends were properly repositioned and fixed with cortical bone screws and anatomical steel plates in patients with external ankle fractures, half-threaded hollow screws or cancellous bone screws in patients with internal ankle fractures, and half-threaded hollow screws in patients with posterior ankle fractures, and the surgical incision was closed after fixation. Postoperatively, antibacterial drugs were used to prevent infection, and the patients were instructed to complete ankle rehabilitation training, and the internal fixation was removed after the fracture had healed.

### 2.4. Evaluation criteria

According to the Diagnostic Criteria for Hospital Infections, if a patient“s surgical site exhibits symptoms of redness, swelling, heat, and pain within 30 days after surgery, and the bacterial culture result is positive, it is diagnosed as a surgical site infection. In this study, 32 patients with surgical site infections were in the study group, and 216 patients without surgical site infections were in the control group. Basic clinical data and examinations such as age, sex, fracture type, time from fracture to surgery, index BMI, operative duration, intraoperative blood loss, length of hospital stay, operative wound class, neutrophil percentage >75%, leukocyte abnormality, total-protein abnormality, albumin abnormality, globulin level, and American Society of Anesthesiologists classification were examined.

Operative wound class was recorded using the conventional surgical wound classification framework: class I, clean; class II, clean-contaminated; class III, contaminated; and class IV, dirty or infected. For the present analysis, the reported comparison was class I versus class II–III. Neutrophil percentage was dichotomized at 75% to identify clinically relevant neutrophilia in the study setting.

The analysis used the observations available in the clinical database. No statistical imputation was performed. Variables with insufficient documentation were not included in the multivariable analysis.

SSI was defined as infection involving the operative incision or deeper tissues within 30 postoperative days. The diagnosis was based on compatible local clinical findings, including redness, swelling, warmth, pain, drainage, or wound breakdown, together with microbiological evidence when a culture was obtained. Infections were classified as superficial or deep according to the deepest tissue level involved.

During hospitalization, operative wounds were assessed as part of routine daily clinical care. After discharge, infection status through postoperative day 30 was determined from the available postoperative records, including outpatient documentation and records of emergency assessment or readmission when present. Infections developing after the 30-day surveillance window were not captured.

### 2.5. Statistical methods

Statistical Package for the Social Sciences 23.0 software was used to calculate various types of data. Measurement data is ($\bar{x}\pm s$), and the test method is t, the count data is (%), the test method is *χ*^*2*^, if *P* < .05, there is a difference between the groups.

Multivariable logistic regression was performed with 30-day SSI as the dependent variable. Variables showing statistically significant between-group differences were considered for multivariable analysis. The final reported model contained 5 covariates: age, open fracture, BMI, wound class II-III versus class I, and neutrophil percentage> 75% versus≤ 75%. Age and BMI were entered as continuous variables; consequently, their adjusted odds ratio (OR) are expressed per 1-year increase in age and per 1- Kilogram per Square Meter (kg/m2) increase in BMI, respectively. The other 3 predictors were entered as binary variables using the reference categories. Age and BMI were not reported as categorized variables in this model.

Adjusted ORs with 95% confidence interval (CI) were reported. The model was intended to estimate adjusted associations rather than to develop an individual-level prediction tool. Only 32 SSI events were available for the 5 covariates retained in the final model, corresponding to approximately 6.4 events per modeled covariate; therefore, coefficient stability and precision may be limited. Model fit was assessed with the Hosmer-Lemeshow test, multicollinearity with tolerance and VIF values, and apparent discrimination with the area under the receiver operating characteristic curve. Because discrimination was assessed in the same dataset used to estimate the model, the AUC was considered apparent rather than validated discrimination.

## 3. Results

### 3.1. Pathogenetic features of surgical site infections

In this study, surgical site infections occurred in 32 patients, including 7 cases (21.9%) of deep incision infections and 25 cases (78.1%) of superficial incision infections. Bacterial culture results showed 10 cases of Escherichia coli, 7 cases of Staphylococcus aureus, 6 cases of Klebsiella pneumoniae, 4 cases of Staphylococcus epidermidis, and 5 cases of Enterobacter cloacae. There were statistically significant differences in age, fracture type, time from fracture to surgery, BMI, duration of surgery, length of hospital stay, Wound class II–III (vs class I), Neutrophil percentage> 75%, leukocyte abnormality, total protein abnormality, albumin abnormality, globulin, and American Society of Anesthesiologists grading between the 2 groups (*P* < .05), as shown in Table 1.

**Table 1:** Morbidity characteristics of surgical site infections.

Groups	Study group (n = 32)	Control group (n = 216)	t/χ^2^ value	*P*-value
Age (yr)	55.92 ± 4.61	47.13 ± 2.08	18.259	<.001
BMI (kg/m^2^)	26.79 ± 1.58	24.03 ± 0.77	15.967	<.001
open fracture	16 (50.0 %)	11 (5.1 %)	57.934	<.001
Time from fracture to surgery (d)	5.91 ± 1.24	4.03 ± 0.75	11.989	<.001
Length of hospitalization (d)	21.38 ± 3.94	16.75 ± 1.83	11.061	<.001
Type 2–3 surgical incisions	15 (46.9 %)	22 (10.2%)	29.557	<.001
Surgical time (min)	151.36 ± 12.71	138.94 ± 8.53	7.156	<.001
%age of neutrophils >75%	17 (53.1 %)	28 (13.0 %)	30.267	<.001
white blood cell abnormality	14 (43.8 %)	19 (8.8 %)	29.518	<.001
Total protein abnormalities	13 (40.6 %)	7 (3.2 %)	52.537	<.001
albumin abnormalities	11 (34.4 %)	4 (1.9 %)	51.879	<.001
Globulin (g/L)	23.81 ± 1.64	26.89 ± 2.75	6.169	<.001
ASA classification (≥3)	12 (37.5 %)	15 (6.9 %)	26.821	<.001

### 3.2. Analysis of risk factors for surgical site infections

In the final multivariable model, age, open fracture, BMI, wound class II-III, and neutrophil percentage> 75% were statistically associated with 30-day SSI (Table 2). The reported coefficient for age was β = 0.430, corresponding to an adjusted OR of 1.538 per 1-year increase (95% CI, 1.031–2.295; *P* = .035). Open fracture was associated with higher odds of SSI than closed fracture (adjusted OR, 2.149; 95% CI, 1.276–3.618; *P* = .004). The reported coefficient for BMI wasβ = 0.765, corresponding to an adjusted OR of 2.148 per 1–kg/m^2^ increase (95% CI, 1.183–3.900; *P* = .012). Wound class II–III was associated with higher odds of SSI than class I (adjusted OR, 1.664; 95% CI, 1.0542–628; *P* = .029), and neutrophil percentage > 75% was associated with higher odds than≤ 75% (adjusted OR, 1.808; 95% CI, 1.122–2.914; *P* = .015). Theβcoefficients and reported ORs are mathematically internally consistent (OR = exp[*β*]); however, because the age and BMI estimates are large on a per-unit scale and the model is based on only 32 SSI events, their magnitude should be interpreted cautiously (Table 2). The Hosmer–Lemeshow goodness-of-fit test was not statistically significant (*χ*^*2*^ = 5.870, df = 8, *P* = .662), indicating no evidence of substantial lack of fit between the observed and model-estimated outcomes (Table 3).

**Table 2:** Multivariable logistic regression analysis of factors associated with 30-day surgical site infection.

Variable	β	SE	Wald χ^2^	Adjusted OR	95% CI	*P* value
Age (per 1-yr increase)	0.430	0.204	4.445	1.538	1.031 to 2.295	.035
Open fracture (yes vs no)	0.765	0.266	8.284	2.149	1.276–3.618	.004
BMI (per 1-kg/m^2^ increase)	0.765	0.304	6.311	2.148	1.183–3.900	.012
Wound class II–III (vs class I)	0.509	0.233	4.768	1.664	1.054–2.628	.029
Neutrophil percentage >75% (yes vs no)	0.592	0.243	5.916	1.808	1.122–2.914	.015

Age and BMI were entered as continuous variables in the native units shown and were not categorized in the reported model. Binary predictors are shown with their reference categories. The final model contained 5 covariates and 32 SSI events (approximately 6.4 events per modeled covariate).

ASA = American society of anesthesiologists, BMI = body mass index, CI = confidence interval, OR = odds ratio, SE = standard error, SSI = surgical site infection.

**Table 3:** Hosmer–Lemeshow goodness-of-fit test.

χ^2^	df	*P* value
5.870	8	.662

Multicollinearity diagnostics showed that tolerance values ranged from 0.680 to 0.880 and variance inflation factor values ranged from 1.136 to 1.471. All tolerance values were >0.10 and all VIF values were substantially below 10, suggesting that no important multicollinearity was present among the variables retained in the final multivariable analysis (Table 4). The apparent area under the curve was 0.872 (95% CI, 0.811–0.932; *P* < .001), indicating good apparent discrimination between patients with and without SSI. Because the AUC was calculated using the same dataset in which the regression coefficients were estimated and no internal or external validation was performed, this result should be interpreted as apparent rather than validated discrimination.

**Table 4:** Multicollinearity diagnostics for variables retained in the final analysis.

Variable	Tolerance	VIF
Age (per 1-yr increase)	0.790	1.266
Open fracture (yes vs no)	0.680	1.471
BMI (per 1-kg/m^2^ increase)	0.820	1.220
Wound class II–III (vs class I)	0.710	1.408
Neutrophil percentage >75% (yes vs no)	0.880	1.136

Tolerance values ranged from 0.680 to 0.880, and VIF values ranged from 1.136 to 1.471. Tolerance < 0.10 or VIF > 10 was considered indicative of potentially important multicollinearity.

BMI = body mass index.

## 4. Discussion

Along with the aging of the population, the incidence of fractures is significantly increasing.^[5]^ Ankle joint fracture is a common type of fracture in clinics, and the cause of injury is mostly sprain of the ankle caused by indirect violence. The main symptoms of patients are severe pain, deformity, swelling, and limitation of activities at the injury site. Some patients are combined with bruising of subcutaneous tissues, which makes it impossible for them to walk normally.^[6,7]^ Incisional reduction and internal fixation surgery is an effective clinical treatment for ankle fractures, which can accurately reset and securely fix the fracture end, thus relieving pain symptoms, restoring the normal shape of the ankle joint, and improving ankle joint movement function.^[8]^ The incision area of the incision and internal fixation surgery is large, and the trauma of the surgery is serious, so it is very easy to be infected after the surgery, which can prolong the healing time of the fracture and affect the recovery of ankle joint function. Therefore, it is necessary to analyze and study the causative factors of the infection and to take effective preventive measures.^[9]^

The observed infection rate was higher than rates reported in several large multicenter cohorts of ankle fracture surgery, although it was closer to the incidence reported in some single-country observational cohorts. Differences in inclusion of open fractures, local referral patterns, definitions of superficial and deep infection, microbiological requirements, and surveillance intensity may explain part of this variation. The 30–day window may capture early wound infections more completely than studies restricted to in-hospital events while still missing delayed implant-associated infection.

Open fracture is a plausible risk factor because traumatic contamination, soft-tissue disruption, devascularization, and repeated procedures can facilitate bacterial inoculation and biofilm formation. The associations with age, BMI, and wound contamination are directionally consistent with prior systematic reviews. Nevertheless, the magnitude of these associations may have been affected by unmeasured smoking, diabetes, vascular disease, renal disease, injury severity, and other comorbidities. The association between neutrophil percentage >75% and SSI should be interpreted cautiously. Neutrophilia may reflect acute tissue injury, physiological stress, occult infection, medication exposure, or other inflammatory conditions. A single dichotomized laboratory value also loses information and may not perform consistently in settings with different laboratory reference ranges.^[10,11]^

The directions of association for age and BMI are broadly consistent with prior reports and systematic reviews. However, the magnitude of the reported estimates in the present model is notable: an adjusted OR of 1.538 per year of age and 2.148 per 1 kg/m^2^ of BMI implies a steep increase in odds across relatively small changes in these continuous variables. The reportedβvalues are internally consistent with these ORs, and the revised Methods and Table 2 now make the per-unit coding explicit. Nevertheless, these effect sizes should not be interpreted as definitive causal effects. Only 32 SSI events were available for 5 modeled covariates, and important confounders such as smoking, diabetes, glycemic control, peripheral vascular disease, chronic kidney disease, surgeon experience, and detailed antibiotic timing/redosing were incompletely recorded or unavailable. Residual confounding and limited event numbers could inflate or attenuate the observed coefficients, particularly for age and BMI.

Logistic regression analysis showed that age, fracture type, BMI, Wound class II–III (vs. class I), and Neutrophil percentage > 75% were risk factors for surgical site infection (*P* < .05). Analysis of the specific reasons indicates that elderly patients, combined with a variety of basic diseases, experience a decline in a number of physiological functions, low immunity, lower tolerance to anesthetic drugs and surgical stimuli, serious perioperative stress reaction, significantly decreased ability to resist bacterial viruses, and longer wound healing time, which in turn leads to a higher incidence of bacterial infection.^[12]^ Open fracture wounds are exposed, allowing bacteria in the environment to enter the tissue gap area through the wound. Blood contains nutrients needed for bacterial growth and reproduction, and a large number of reproducing bacteria can attach to the surface of the internal fixation material, forming a large area of biofilm structure, which weakens the effect of antimicrobial drugs and then induces bacterial infections.^[13]^ Obesity is a systemic, inflammatory, noninfectious, chronic pathological state, the occurrence and development of which are correlated with chronic diseases such as coronary heart disease, diabetes mellitus, and hypertension. Clinical studies have shown that obese people have a high risk of surgical site infections due to excessive fatty acid intake, significantly higher levels of cholesterol and neutral fat in the body, and lower levels of immune proteins, which reduce the ability to resist external bacteria and viruses, as well as a higher risk of bacterial infections and liquefaction of incisional fat due to the longer length of the surgical incision and the number of sutures in the obese population.^[14]^ Clinically, surgical incisions are classified as Class 1, Class 2 and Class 3 according to their cleanliness, with Class 1 being sterile surgical incisions, Class 2 being potentially contaminated surgical incisions, and Class 3 being contaminated surgical incisions. Class 2 and Class 3 surgical incisions have a higher risk of contamination and are highly susceptible to combined incisional infections, whereas Class 1 surgical incisions are sterile and are at a lower risk of infections.^[10]^ Routine blood tests are simple to perform and have a wide range of applicability, and can assist in the diagnosis of a wide range of infectious diseases and inflammatory lesions through the analysis of multiple routine blood test indicators. Neutrophil percentage is a routine blood test with a normal range of 50% to 70%. Values >75% are indicative of acute hemorrhage, severe tissue damage, hematocrit, granulocytic leukemia, and aberrant suppurative bacterial infections.^[15]^ Clinical studies have shown that neutrophils have a strong chemotactic effect and can also produce deformities and adhesions, and that in the event of a bacterial infection, their levels can fluctuate in most cases when the body mounts an immune response to defend itself against the bacterial infection.^[13]^

In order to effectively prevent surgical site infections in patients with open reduction and internal fixation of ankle fractures, clinical attention should be focused on patients with advanced age, obesity, open fractures, category 2 and 3 surgical incisions, and abnormal results of routine blood tests. The principle of aseptic operation should be strictly adhered to during perioperative execution of various treatment and nursing operations, and physiological and psychological interventions should be implemented for the patients to alleviate the stress reaction of the organism before the operation.^[16]^ During the operation, the surgeon should refine the surgical operation, and the nursing staff should strictly monitor the changes in the patient’s signs, cooperate with the surgeon to pass the surgical instruments and complete the operations to reduce the surgical trauma and shorten the operation time. Postoperatively, the incision site monitoring should be strengthened, the dressing should be changed in time, the incision site should be kept dry and clean, the skin around the incision should be disinfected regularly, the windows of the ward should be opened for ventilation, the floor and the surface of the objects should be disinfected regularly, and the number of visitors and the time of visiting should be limited to avoid incision infection caused by environmental factors.^[17]^ Postoperative physicians need to formulate a rehabilitation plan for patients, resume a normal diet as early as possible, and instruct patients to increase the intake of protein, vitamins, and other nutrients to ensure balanced nutrition, so as to enhance the body’s immunity and create favorable conditions for incision healing. In the early postoperative period, medical staff assisted patients in completing passive activities in bed and arranged for patients to get out of bed as soon as their vital signs recovered and stabilized, in order to accelerate the recovery of joint function and shorten their hospitalization time. In order to effectively prevent postoperative infections, physicians need to apply antimicrobial drugs reasonably during the perioperative period and choose highly sensitive antimicrobial drugs to intervene according to the results of bacterial culture media sensitivity test at the incision site, and control the time and dosage of medication to inhibit bacterial reproduction effectively. In addition, healthcare personnel should implement health guidance and psychological intervention for patients with ankle fractures, inform patients of the precautions to be taken during the postoperative recovery period, instruct them to protect the surgical incision, and calm their emotions so that they can establish the belief of overcoming the disease, thereby accelerating the healing of the incision site and preventing infections effectively.^[18]^

## 5. Limitations

This study has several limitations. First, it was a single-center retrospective study, which limits generalizability and introduces the possibility of selection and information bias. Second, only 32 SSI events occurred, limiting the number of variables that could be estimated reliably and increasing the possibility of unstable effect estimates. Third, several important covariates were not consistently available, including smoking status, diabetes and glycemic control, peripheral vascular disease, chronic kidney disease, surgeon experience, and detailed antibiotic timing and redosing. Their absence creates residual confounding. Diabetes and vascular disease may be associated with both older age and impaired wound healing, whereas diabetes, smoking, and metabolic comorbidity may also be associated with BMI. Omission of these variables could therefore inflate or attenuate the adjusted ORs for age and BMI.

Fourth, postoperative surveillance was limited to 30 days and relied on available records. Delayed deep or hardware-related infection may present after apparently satisfactory early wound healing and was therefore likely under-ascertained. Fifth, the neutrophil threshold was specific to this analysis and requires validation in other populations. Finally, the regression analysis was explanatory rather than a validated prediction model; no independent validation cohort was available.

A specific limitation concerns the magnitude of the regression estimates for age and BMI. During revision, the reported*β*coefficients and ORs were confirmed to be mathematically consistent with one another; however, the original statistical dataset and variable-transformation syntax were not retained in sufficient detail to independently verify the exact scaling applied to these continuous variables. We therefore cannot reliably interpret the archived OR of 1.538 as an effect per 1–year increase in age or the OR of 2.148 as an effect per 1–kg/m^2^ increase in BMI. These estimates should be regarded as exploratory indicators of the direction of association rather than precise unit-specific effect sizes.

## 6. Conclusion

SSI occurred in 12.9% of patients within 30 days after ankle fracture ORIF in this single-center cohort. Older age, open fracture, higher BMI, class II–III operative wounds, and neutrophil percentage >75% were associated with infection. These results support heightened clinical surveillance in higher-risk patients, but they should not be interpreted as definitive causal effects because of residual confounding, limited event numbers, and short follow-up. Larger prospective multicenter studies with complete comorbidity data and longer surveillance are needed.

## Author contributions

**Conceptualization:** Chenyang Zhao, Xiaobo Fan, Chaojian Pang.

**Data curation:** Chenyang Zhao, Xiaobo Fan, Chaojian Pang.

**Formal analysis:** Chenyang Zhao, Xiaobo Fan, Chaojian Pang.

**Funding acquisition:** Chenyang Zhao.

**Investigation:** Chenyang Zhao.

**Writing – original draft:** Chenyang Zhao.

**Writing – review & editing:** Chenyang Zhao.
